# Biochemical Indices and Life Traits of Loggerhead Turtles (*Caretta caretta*) from Cape Verde Islands

**DOI:** 10.1371/journal.pone.0112181

**Published:** 2014-11-12

**Authors:** Sara Vieira, Samir Martins, Lucy A. Hawkes, Adolfo Marco, M. Alexandra Teodósio

**Affiliations:** 1 Centro de Ciências do Mar, Universidade do Algarve, Campus de Gambelas, Faro, Portugal; 2 University of Exeter, College of Life and Environmental Sciences, Penryn Campus, Cornwall, United Kingdom; 3 Estación Biológica de Doñana (CSIC), Américo Vespucio, Sevilla, Spain; University of South Dakota, United States of America

## Abstract

The loggerhead turtle (*Caretta caretta*) is an endangered marine reptile for whom assessing population health requires knowledge of demographic parameters such as individual growth rate. In Cape Verde, as within several populations, adult female loggerhead sea turtles show a size-related behavioral and trophic dichotomy. While smaller females are associated with oceanic habitats, larger females tend to feed in neritic habitats, which is reflected in their physiological condition and in their offspring. The ratio of RNA/DNA provides a measure of cellular protein synthesis capacity, which varies depending on changes in environmental conditions such as temperature and food availability. The purpose of this study was to evaluate the combined use of morphometric data and biochemical indices as predictors of the physiological condition of the females of distinct sizes and hatchlings during their nesting season and how temperature may influence the physiological condition on the offspring. Here we employed biochemical indices based on nucleic acid derived indices (standardized RNA/DNA ratio-sRD, RNA concentration and DNA concentration) in skin tissue as a potential predictor of recent growth rate in nesting females and hatchling loggerhead turtles. Our major findings were that the physiological condition of all nesting females (sRD) decreased during the nesting season, but that females associated with neritic habitats had a higher physiological condition than females associated with oceanic habitats. In addition, the amount of time required for a hatchling to right itself was negatively correlated with its physiological condition (sRD) and shaded nests produced hatchlings with lower sRD. Overall, our results showed that nucleic acid concentrations and ratios of RNA to DNA are an important tool as potential biomarkers of recent growth in marine turtles. Hence, as biochemical indices of instantaneous growth are likely temperature-, size- and age-dependent, the utility and validation of these indices on marine turtles stocks deserves further study.

## Introduction

The impact of anthropogenic activity on biodiversity has now been demonstrated throughout terrestrial and aquatic ecosystems [1], [2]. Impacts include, but are not limited to, historic and ongoing harvest (legal and illegal) [3], accidental take (e.g. fisheries by-catch) [4], habitat destruction and degradation [5] and climate change [6]. In assessing recovery from such impacts to inform conservation management, it is imperative to gain information on population growth rate and health status. Life history traits determine the schedule and duration of key events in an organism's lifetime that are shaped by natural selection to produce the largest possible number of surviving offspring. The ratio of RNA to DNA (known as ‘standardized RNA/DNA ratio’- sRD) has been widely used as a biochemical index to assess potential survival and growth and in determining the ecophysiological condition of marine organisms [7]. This index has quickly become a particularly promising biochemical tool that not only reflects physiological condition, but also allows for the estimation of instantaneous growth rates in a great variety of organisms [8], [9], [10], [11].

The usefulness of sRD as a measure of physiological condition is related to the fact that the concentration of cellular DNA is relatively constant in the somatic cells regardless of any changes in the organism's environment, while the RNA content of a cell increases as the cellular demand for protein synthesis and growth increases [7]. The ratio of RNA/DNA thus provides a measure of cellular protein synthesis capacity, which varies depending on changes in environmental conditions such as temperature and food availability. Poor nutritional condition contributes to low protein synthesis and slow growth, resulting in a low RNA/DNA ratio [9], [10]. Consequently, individuals in good nutritional condition generally have high levels of RNA/DNA, whereas individuals undergoing dietary restriction have a lower amount of RNA in their cells and hence a lower rate of RNA/DNA [12].

Despite its widespread use in marine fishes and invertebrates, sRD has mainly been validated for application to studies with small marine organisms. The application of this method in studies with large migratory organisms, such as marine vertebrates, should offer important insights, particularly in view of the conservation concern for most marine vertebrate taxa [7]. Moreover, sRD yields specific information about the physiological condition and growth rate of animals in the different areas they occupy throughout their life cycle, with a minimal amount of distress to the animal.

Of the marine megavertebrates, marine turtles are one of the best studied taxa, with all seven species having now been tracked worldwide [13], [14]. However, management of sea turtles is still hindered by a lack of key data on turtle biology, population status and environmental threats [15]. The loggerhead sea turtle, *Caretta caretta*, is an endangered species with a circumglobal distribution. Human exploitation of this species over the last few centuries has led to dramatic population declines [16]. Assessing the current status and predicting the viability of wild populations of marine turtles requires the quantification of demographic parameters such as individual growth rate [17], [18]. However, gathering such growth rates for marine turtles typically involves mark and recapture programs in which recapture probabilities can be quite low [19] or repeated sampling of a population for use in length-frequency analyses [20].

Satellite tracking has now demonstrated that many populations of marine turtles exhibit plasticity with multiple foraging strategies seen in populations of adult turtles. For example, in loggerhead turtles in some areas, larger adult turtles may be more likely to forage in shallow neritic habitats, whereas smaller adult turtles appear to be more likely to forage in oceanic habitats [21], [22], [23], [24], [25], [26]. It is not yet understood whether these differences in size are related to differences in age, as oceanic and neritic foragers have been suggested to reach sexual maturity at similar ages [23]. As differing strategies may be expected to have different growth rates and nutritional status, loggerhead sea turtles provide an excellent model system with which to test this methodological approach on a marine vertebrate species.

Establishing techniques with which to supplement mark-recapture and length-frequency analyses for estimating recent growth rates of turtles upon first capture would also substantially improve our ability to evaluate the status of loggerhead turtle populations on a shorter timescale. In addition, loggerhead turtles expend considerable energy migrating from foraging areas to breeding and nesting areas. Loggerhead turtles nest on average every 12 to 17 days during the breeding season and return to breed every 2 to 4 years, presumably because the energetic cost of doing so prohibits breeding more frequently [27]. It seems that food availability and regional or temporal variations may limit growth rate and physiological condition, which may be reflected in breeding frequency and on the fitness of offspring [28]. The purpose of this study was to evaluate the combined use of morphometric, behavioral data and biochemical indices based on nucleic acid derived indices (*e.g*., standardized RNA/DNA ratio-sRD, RNA concentration and DNA concentration) as predictors of the physiological condition of the females and hatchlings during their nesting season, and to assess how temperature may influence the physiological condition of offspring. We suggest this may help towards development of minimally invasive growth and viability measurement strategies, which are essential in planning future conservation strategies.

In this study, we aimed to investigate the following hypotheses: regarding adult turtles, (i) Does the physiological condition of females decrease during the nesting season (A1)?; (ii) Is the physiological condition of “large” adult sea turtles associated with neritic habitats different to the physiological condition of “small” adult sea turtles associated with oceanic habitats (A2)? Regarding hatchling sea turtles, (i) Are the hatching and emergence successes of “small” adult sea turtles lower than in “large” adult sea turtles (O1)?; (ii) Are hatchlings produced by “large” females larger than the hatchlings produced by “small” females (O2)?; (iii) Is hatchling vigour correlated with physiological condition (O3)?; and (iv) Does incubation temperature influence the physiological condition of the hatchlings (O4)?

## Methods

### Study area

**Figure 1:**
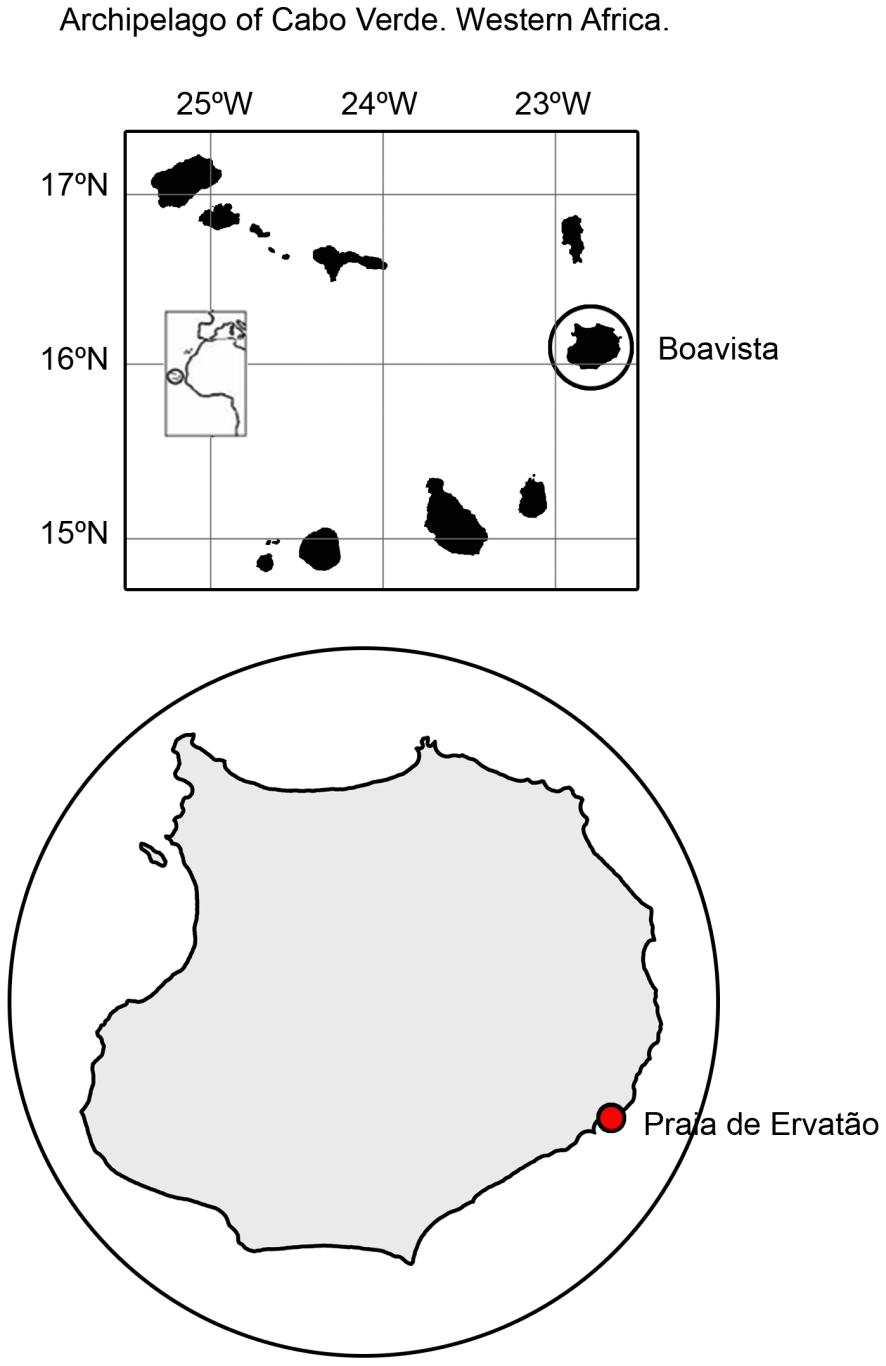
Map of the Cape Verde Archipelago, Boavista Island (Republic of Cape Verde, western Africa) and the study site (framed area).

The Cape Verde Islands are located in the eastern North Atlantic between 14° 45′ and 17° 18′ N and 22° 38′ and 25° 22′ W, 500 km off the coast of Senegal (Fig. 1). The local weather conditions give this tropical region a moderate subtropical climate. The loggerhead nesting season in Cape Verde is prolonged, extending from June through October. This volcanic archipelago hosts the third largest rookery of loggerhead turtles in the world [29] and the population is reproductively isolated from the other Atlantic loggerhead rookeries [30]. Furthermore, the Cape Verde population constitutes the most endangered regional management unit for this species in the Atlantic [31].

### Ethics Statement

The present study was carried out on Ervatão beach, within the *Reserva Natural da Tartaruga*, which is included in the national protected area network of Cabo Verde (Decreto-Regulamentar n° 14/2013 de 9 de Maio), operated by the “Direcção Geral do Ambiente”, an agency of the Cape Verdian Ministry of the Environment. The work was ethically approved by the “Direcção Geral do Ambiente” and loggerhead sea turtle tissues were imported to Portugal (University of Algarve) under a CITES permit.

### Sampling and data collection

We collected a total of 57 tissue samples from the right front flipper from randomly selected nesting females during the beginning (7th to 12th of July) and end (19th of September to 10th of October) of the 2012 nesting season from one of the main nesting beaches, Praia Ervatão. The skin tissue samples (<2 mg) were taken using a 5-mm biopsy punch, while the female was covering her completed nest with sand. We stored samples in a RNAlater solution at 4°C. Nesting females were measured (curved carapace length: CCL_min_) using a fiberglass tape measure (±0.1 cm) and turtles classified as “small” (≤85 cm CCL_min_; 55% of turtles; n = 31) and “large” (>85 cm CCL_min_; 45% of the total; n = 26; based on neritic/oceanic separation by Eder *et al.* 2012 [32]). Nests laid by females at the beginning of the season were immediately translocated to an artificial hatchery, taking care to maintain the original vertical orientation of the eggs during transport, and reburied at 40 cm depth (mean nest depth for Boavista [25]). The artificial hatchery (50×15 m^2^) was established on Benguinho Beach, a natural nesting beach area adjacent to Praia Ervatão.

**Figure 2:**
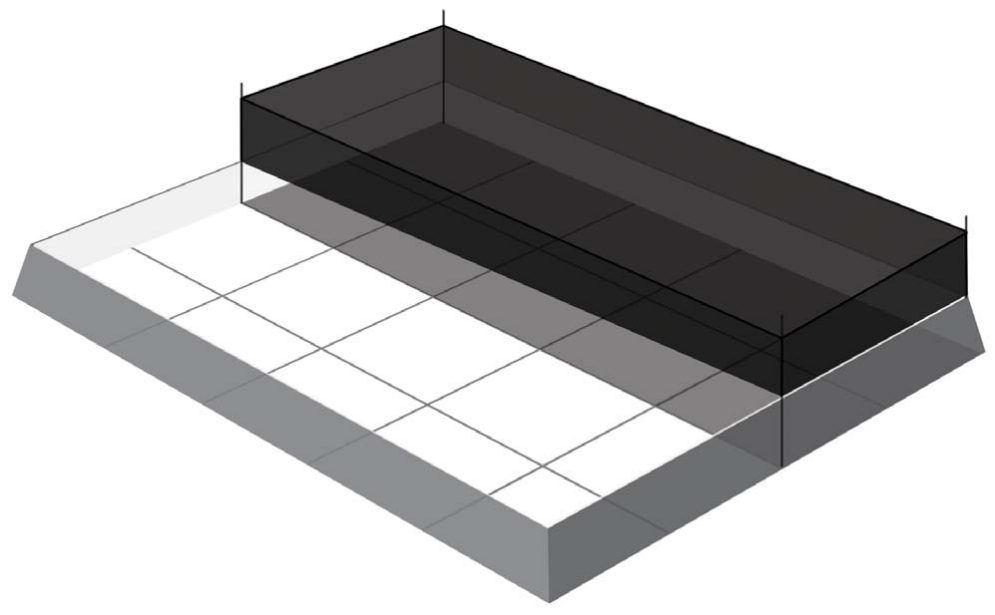
Diagram of experimental design illustrating the two experimental treatments: shaded (under a shade cloth made of plastic mesh) and unshaded (uncovered nests).

In order to investigate if the temperature might influence hatchling physiological condition (as measured using sRD) we manipulated nest temperature as follows: We marked and randomly assigned the 20 nests to one of two experimental treatments: either shading them under a shade cloth made of plastic mesh over the sand surface above the nests (‘shaded’; n = 10); or leaving them uncovered (‘unshaded’; n = 10) (Fig. 2). We assumed that shading significantly lowered nest temperatures [32], by reducing solar infra-red radiation, and arranged nests in parcels of 1 m^2^ for every nest, distributing nests of different treatments in a block design of five nests per treatment block. We placed a round plastic net (45 cm in diameter, 50 cm in height) over all nests 45 days after egg-laying to collect hatchlings after emergence. Nests were checked throughout the night and again at daybreak to check for hatchling emergence. Within two hours of emergence, we measured hatchling straight carapace length (SCL) with a digital caliper (±0.1 mm), assessed hatchling vigour and collected tissue samples from 3 randomly selected hatchlings from each nest. To assess hatchling vigour we placed hatchlings (n = 10 per nest) on their dorsal side and the time taken for hatchlings to ‘right’ themselves was recorded [33]. The skin samples (<1 mg) were taken on a sanitized polyethylene board (10×10 cm^2^) with one front flipper held flat against the board and isopropyl alcohol applied to disinfect the biopsy site. A single sample was then taken using a 2-mm biopsy punch (with plunger) from the trailing edge of the front flipper proximal to the body out to midway along the flipper [35]. Handling time did not exceed 15 minutes. Hatchlings were released immediately after the sampling event.

### Determination of nucleic acids, concentrations and ratios

Nutritional condition was assessed using the following nucleic acid acid-derived indices: sRD (standardized RNA/DNA ratios), DNA/mg and RNA/mg per tissue. Nucleic acid analysis was carried out for ecophysiological condition of breeding females in the beginning and at the end of the breeding season and for hatchling condition at the shaded/unshaded treatments. The procedures used to quantify nucleic acids in individual females and hatchlings are outlined in Caldarone *et al.* (2001) [36] and Esteves *et al.* (2000) [37] using muscle tissue [38]. Briefly, tissues were mechanically and chemically homogenized and subsequent fluorescence-photometric measurements were taken using ethidium bromide (EB) as a specific nucleic acid fluorochrome dye. Fluorescence was measured on a microplate reader (Biotek synergy HT model SIAFRTD) using an excitation wavelength of 365 nm and an emission wavelength of 590 nm. We measured endogenous fluorescence (before EB addition) from the first set of samples from each tissue, but this was found to be negligible, so it was disregarded in the calculations of nucleic acid concentrations. Total fluorescence was first read, and then samples were incubated with ribonuclease A (Type-II A) at 37°C for 30 minutes, and then cooled to room temperature before reading. The fluorescence due to total RNA, mainly ribosomal, was calculated as the difference between total fluorescence (RNA and DNA) and the fluorescence measured after ribonuclease treatment, which is assumed to be due to DNA. Concentrations were determined by running standard curves of DNA–EB (Ethidium bromide) and RNA–EB with known concentrations of u-phagus DNA (0.25 ug uLl–1) and 16S–23S s *E. coli* RNA (4 ug uLl–1) (Roche), in the appropriate range of values. The average ratio of DNA and RNA slopes (mean ± SE) was 2.84±0.20 SE. RNA/DNA ratios were standardized (sRD) using this information and the reference slope ratio of 2.4, according to Caldarone *et al.* (2006) [39].

### Data Analyses

All graphics and statistical analyses were performed using the open source software R version 3.0.0 (R Development Core Team; www.r-project.org).

Data were analysed using linear models, after removal of statistical outliers and testing for normality, and the influence of independent factors (environmental/behaviour; beginning/end of breeding season, large/small female, shaded/unshaded) and independent variables (time to righting) on dependent variables (ecophysiological condition of hatchlings and adult females based on sRD ratio, size, and hatching success) were analyzed. We also performed two regression analyses to investigate the relationship among the ecophysiological condition of hatchlings and the hatching success of their nests and their righting time. Significance was assigned at p<0.05.

## Results

### Adult females

**Figure 3:**
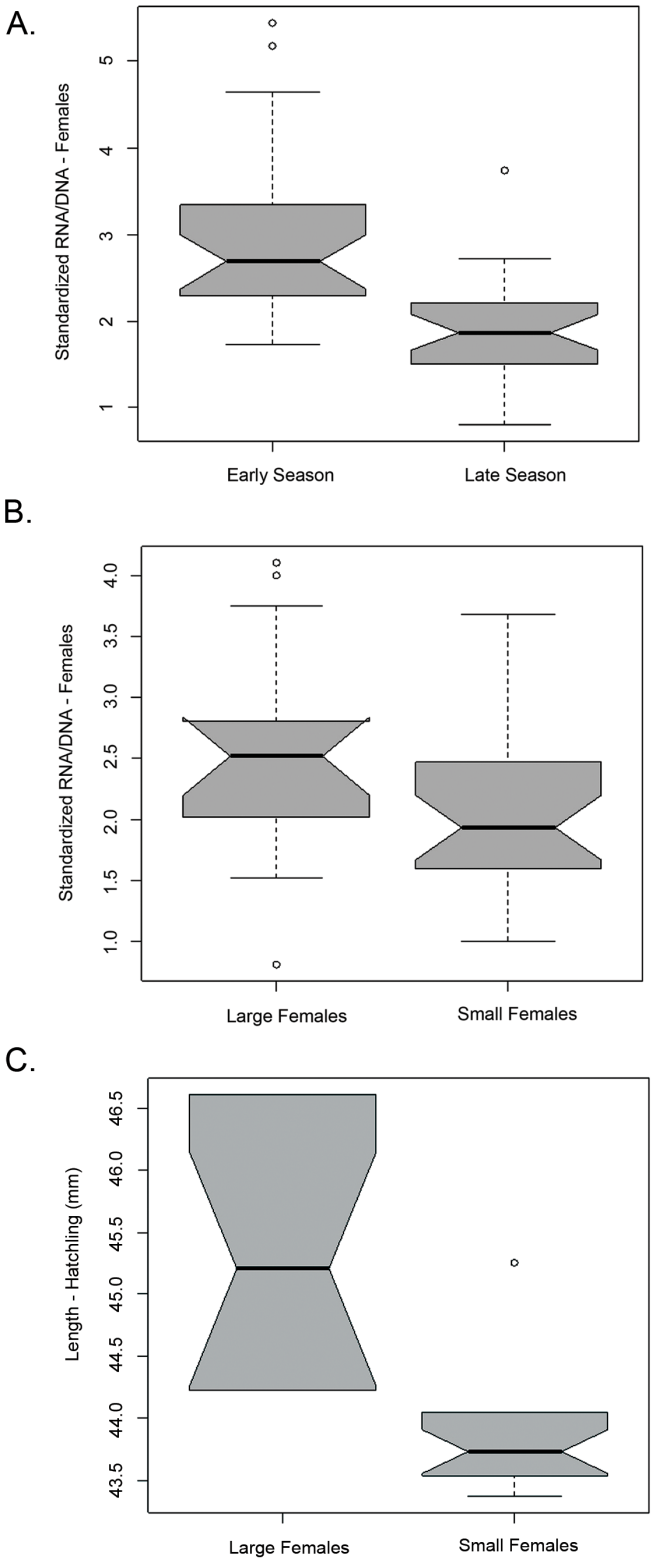
Biochemical condition of sea turtles and Hatchling length. (**A**). Standardized RNA/DNA ratio (mean ± standard error) for sea turtles in the beginning of the season (Early Season, n = 28) (7^th^–12^th^ of July) and at the end of the season (Late Season, n = 29) (19^th^ of September–10^th^ of October); (**B**). Standardized RNA/DNA ratio (mean ± standard error) for “large” sea turtles (CCL_min_>85 cm, n = 26) and for “small” sea turtles (CCL_min_≤85 cm, n = 31); (**C**). Hatchling carapace length (mean ± standard error) for “large” sea turtles (CCL_min_>85 cm) and for “small” sea turtles (CCL_min_≤85 cm).

Ecophysiological condition of nesting females was higher at the beginning of the season (sRD = 2.9±0.95) compared to the end of the season (sRD = 1.9±0.98) (F_1,45_ = 23.4, p<0.001; Fig. 3A). Nesting females from the beginning of the season also exhibited higher RNA and DNA concentrations than nesting females sampled at the end of the season (F_1,55_ = 25.95, p<0.001; F_1,55_ = 7.70, p = 0.008, respectively) such that females sampled at the beginning of the season ([RNA] = 17.11±9.52, [DNA] = 7.10±3.94) had more than the double the RNA concentration than the females sampled at the end ([RNA] = 6.51±9.21, [DNA] = 4.23±4.06).

Although RNA concentration was not significantly different for small and large females (CCL_min_≤85 cm and >85 cm respectively) (F_1,43_ = 0.81, p = 0.37), the analysis of sRD for the two distinct size groups reveal significant differences for the biochemical index (Table 1, Fig. 3B).

**Table 1:** Results of one way-ANOVA for hypotheses in the present study: (A1) Does the physiological condition of females decrease during the nesting season?; (A2) Is the physiological condition of “large” adult sea turtles associated with neritic habitats different to the physiological condition of “small” adult sea turtles associated with oceanic habitats?

Hypothesis	ANOVA	SS	DF	F-stat	p
A1	Time of season	14.020	1	23.4	1.59e-5***
	Residuals	26.979	45		
A2	Length Female	2.20	1	3.919	0.05.
	Residuals	23.019	41		
O1	Hatching Sucess	0.064	1	1.020	0.32
	Residuals	3.281	52		
O2	Length Hatchling	17.093	1	23	2.964e^−5^***
	Residuals	26.006	35		
O3	Time Righting	321.589	1	3.923	5e^−45^***
	Residuals	55.628	52		
O4	Shading	16.8	8	11.12	2.813e^−11^***
	Residuals	19.824	105		

Regarding hatchling sea turtles, (O1) Are the hatching and emergence successes of “small” adult sea turtles lower than in “large” adult sea turtles?; (O2) Are hatchlings produced by “large” females larger than the hatchlings produced by “small” females?; (O3) Is hatchling vigour correlated with physiological condition?; and (O4) Does incubation temperature influence the physiological condition of the hatchlings? DF- degrees of freedom; SS- sum of squares; F-stat - F-statistic; p- significance value (Significant codes: 0 ‘***’ 0.001 ‘**’ 0.01 ‘*’ 0.05 ‘.’).

### Hatchling turtles

**Figure 4:**
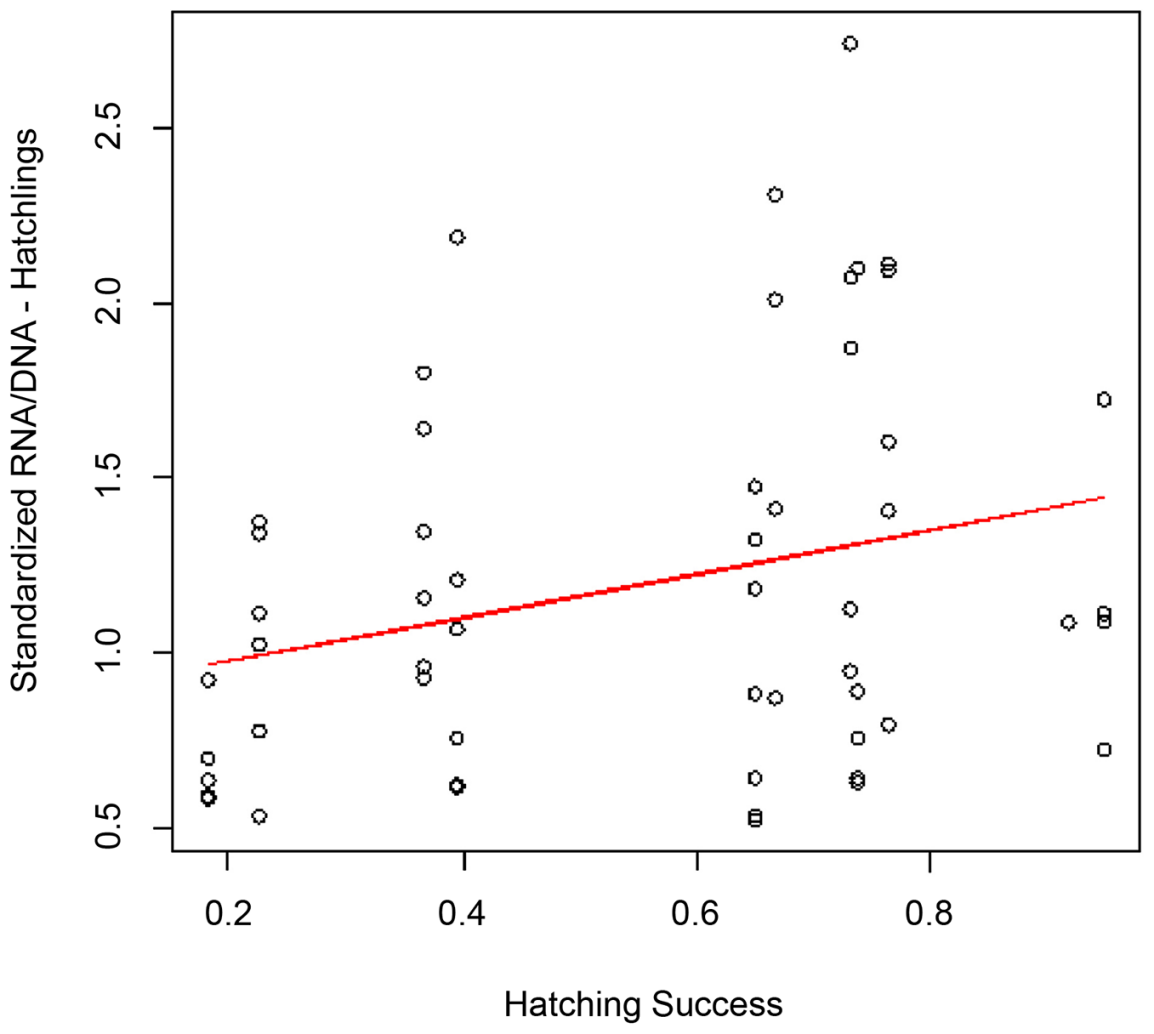
Relationship between the average condition of hatchlings (standardized RNA/DNA ratio) and the hatching success of the nest they came from (R^2^ = 0.06, p<0.001, F_1,52_ = 4.28, p = 0.04).

Hatching success of nests laid by “small” turtles was not significantly different to that of “large” turtles (Table 1). However, hatchlings produced by “large” females were significantly larger than those produced by “small” females (F_1,35_ = 23, p<0.001; Table 1, Fig. 3C). Although hatching success was not correlated with the size of the nesting females, hatching success was positively correlated with the ecophysiological condition of the hatchlings (sRD) (F_1,52_ = 4.282, p<0.05) (Fig. 4).

**Figure 5:**
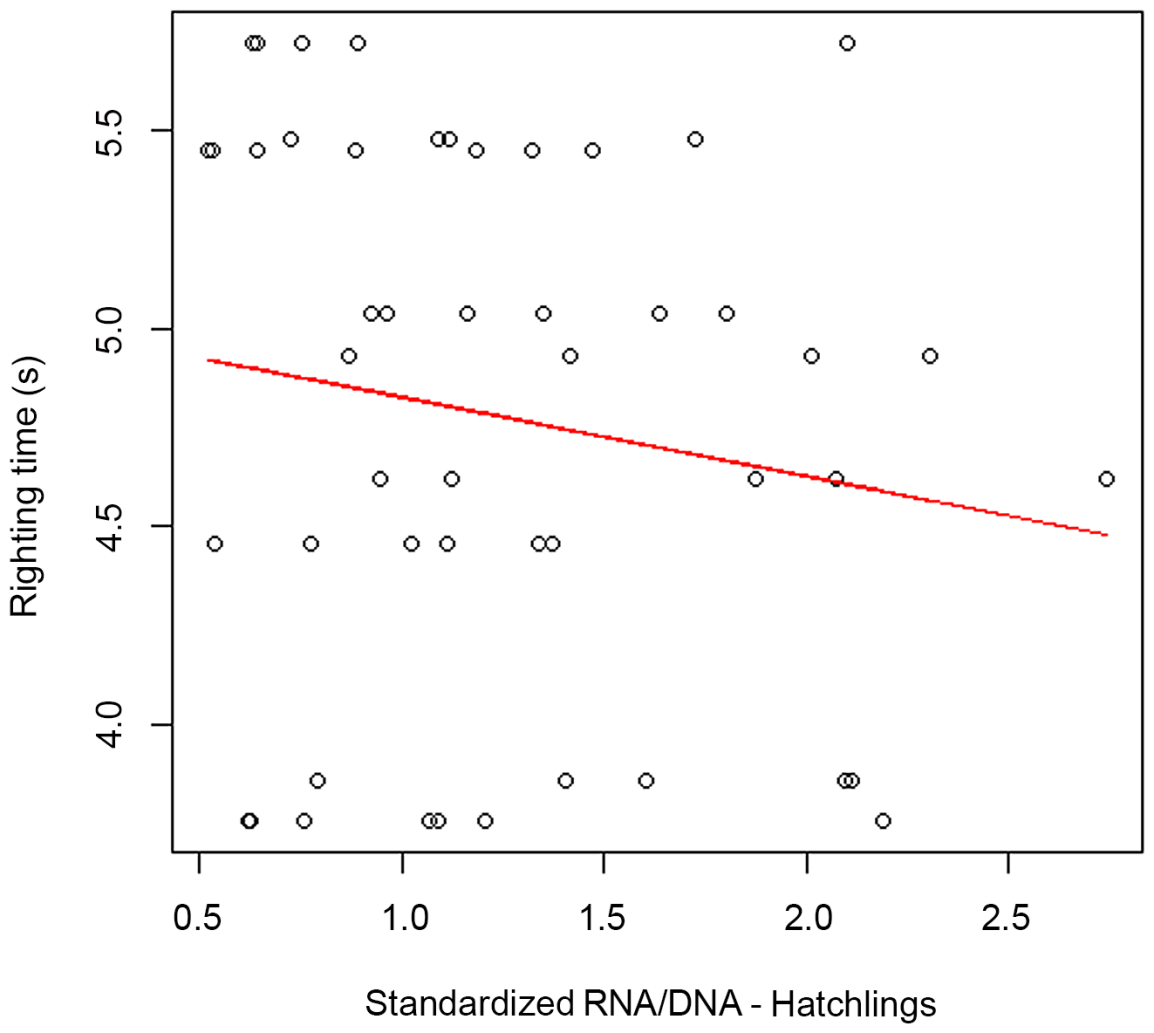
Relationship between the average condition (standardized RNA/DNA ratio) of the hatchlings and their righting time (R^2^ = 0.077, p<0.0001, F_1,52_ = 3.923, p<0.0001).

The ecophysiological condition of the hatchlings (sRD) was negatively correlated with righting time (F_1,52_ = 3.932,p<0,0001; Fig. 5) such that hatchlings with a lower average condition (sRD) took significantly longer to right (Table 1).

### Unshaded versus Shaded nests

**Figure 6:**
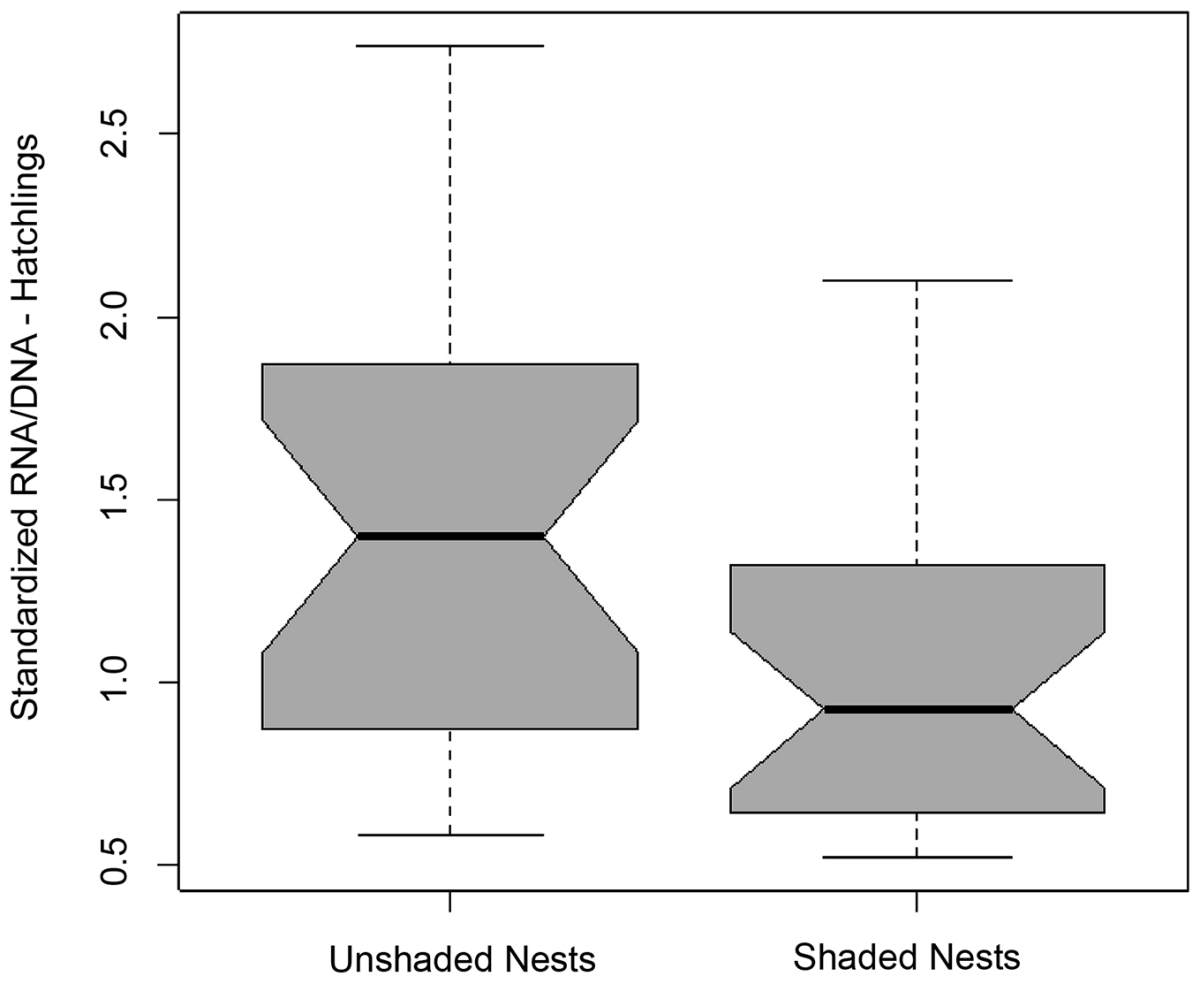
Standardized RNA/DNA ratio (mean ± standard error) for hatchlings from shaded and unshaded nests.

Although hatchlings from shaded nests exhibited higher DNA concentration (mean ± s.d. = 3.27±2.85) than those from unshaded nests (2.76±2.75) and hatchlings from unshaded nests had a higher average RNA concentration (3.19±2.77) than hatchlings from shaded nests (3.03±2.86), they were not statistically significantly different (F_1,56_ = 0.50, p = 0.48; F_1,56_ = 0.05, p = 0.8). On average, hatchlings from unshaded nests had a higher sRD (1.39±0.75) than the hatchlings from shaded nests (0.9±0.5) (F_8,105_ = 11.12, p<0.001; Table 1, Fig. 6).

## Discussion

The major finding of our research was to demonstrate that physiological condition of females decrease during the nesting season (Hypothesis A1). In fact, the purpose of this study was to evaluate the ecophysiological condition of loggerhead turtles using novel and cost effective biochemical behavioral indices to infer life history traits. We were able to support the utility of this approach using a population for which a strong dichotomy in foraging ecology is known to exist (Hypothesis A2). Validation of assays for this method with substantial predictive power for estimating growth rates would provide a less invasive alternative to mark and recapture programs for marine vertebrates and would feed into monitoring programs for populations of endangered species. This study represents the first application of tissue nucleic acid content analysis to evaluate the physiological condition or potential growth of loggerhead sea turtles.

Loggerhead turtles gather the energy necessary for reproduction over several years while in their foraging areas, before they migrate to mate and nest. Loggerhead turtles return to beaches in the region in which they hatched (termed ‘philopatry’), typically after intervals of two to four years (although much longer intervals have been recorded), thought to depend in part on the quality and quantity of food available in foraging areas [27], [28]. Marine turtles likely consume little food during the migration and nesting period and they breed, on average every 12 to 17 days during the breeding season [28]. A substantial decrease in food intake during the nesting season is expected to influence biochemical parameters, including RNA and DNA concentrations. In the present study, nesting females from the beginning of the nesting season exhibited higher nucleic acid concentrations and better physiological condition (sRD) than the females sampled at the end of the season. Similar findings have also been reported in leatherback turtles [40], hawksbill turtles [41] and green turtles [42]. In fact, marine turtles rely on the mobilization of fat stores during the nesting season, which is supported by a large decline in plasma triglycerides [42]. Perraut *et al.* (2014) [40] found that gravid leatherbacks may experience protein loss during the nesting season. This trend is also common in birds, where total protein decreases during egg production [44].

Meyer *et al.* (2012) [45] analysed the starvation-induced changes in biochemical condition in early life stages on 9 species of marine fishes. In all cases, the mean biochemical condition (sRD) decreased exponentially with starvation time, regardless of their initial condition. In addition, they demonstrated that muscle growth during feeding was highly correlated with sRD because of its high protein synthesis rate. Therefore, a starvation signal can be strongly expressed in muscle tissue even though it only accounts for a part of the physiological response to resource limitation.

Larger nesting females exhibited a higher biochemical index (sRD) than “small” nesting females, which may mean that large females have a higher growth potential, due to differences in their diets. Adult female loggerhead sea turtles show a size-related behavioural and trophic dichotomy within several populations: oceanic planktivory by small females and neritic benthivory by large females [21], [22], [23], [24], [25]. Although oceanic adult females are more prevalent in Cape Verde [24], adult neritic foragers apparently have better fitness, as evidenced by larger carapace length, larger clutch size and higher reproductive success [21], [32], [46].

Although “large” nesting females exhibited a higher growth potential than smaller nesting females, there were no significant differences in hatching or emergence success (Hypothesis O1). This may suggest that egg quality was similar between the two foraging groups [47] but the findings of the present study differ from Eder *et al.* 2012 [32], who showed that clutch volume was higher in neritic turtles than oceanic type turtles and was significantly correlated with trophic foraging level, as indicated using stable isotope ratios. This latter result may have been due to the fact that we used relocated nests, which may have a drastic effect on hatching success [48], [49].

Buskirk & Crowder (1994) [50] hypothesized that loggerhead sea turtles that experience a nutritional advantage during development may grow large and realize their increased reproductive potential by laying larger eggs and producing larger hatchlings. Although we did not compare egg size among large neritic foraging and small oceanic foraging females, our results showed that hatchlings produced by “large” females were significantly larger than those produced by “small” females (Hypothesis O2). Eder *et al.* (2012) [32] noted that body size of nesting loggerhead turtles at Cape Verde was not correlated with the sizes of their eggs, but was with the body size of their hatchlings, contrary to some previous studies [47], [51], [52]. Theoretically, offspring size is considered to be an important determinant of fitness in many reptiles, and larger size may have several advantages. The larger size may allow hatchlings to escape gape-limited predators, swim faster, and to successfully handle larger prey items [53], [54], which may lead to enhanced survival during development [55]. Conditions during incubation and emergence are thought to have a significant effect on the size and locomotor performance of turtle hatchlings [56]. In the present study, hatchlings exhibiting a lower ecophysiological condition (sRD) took longer to right themselves than hatchlings with a higher sRD (Hypothesis O3). On land, sea turtle locomotion appears to be inefficient and, as a result hatchlings may overturn or become trapped by marine debris [57]. If they remain on beaches for long periods, particularly during the day, hatchlings may experience an increased risk of desiccation, overheating from sun exposure and predation [58]. Therefore, the ability of hatchling sea turtles to right themselves is critical. The present study shows that hatchlings with a better physiological condition have a better chance of surviving the journey from nest to sea. Additionally, the righting response has been considered as an indicator of fitness in freshwater turtles [34], [58].

The results of the present study also indicate that shaded nests produce hatchlings of lower ecophysiological condition (Hypothesis O4). Other studies have suggested that temperature is one of the main factors with the potential to influence reproductive fitness in marine turtles, as well as a number of phenotypic traits, including hatchling sex [28]. For example, Booth *et al.* (2004) [59] showed incubation temperature influenced hatchling fitness in green sea turtles such that cooler nests produced males with inferior swimming ability compared with warmer nests that produced females, which may be critical during the ‘hatchling frenzy’ (the first 30–60 min of swimming in the shallow water surrounding natal beaches, where predation is highest) [54]. Therefore, it may not be surprising that nesting beach sand temperatures from the study population and from other Atlantic populations suggest a female-biased hatchling sex ratio [59], [60]. Understanding the micro-environmental factors that influence hatching success and hatchling survival can further our knowledge of the habitat requirements for successful sea turtle reproduction.

Nucleic acid concentrations and ratios have become an important tool as biomarkers of recent growth in fish [9], crustaceans [8], bivalves [11] and plankton [10]. Combining morphometric measurements with biochemical analyses of skin and other easily obtainable tissues (including blood) could provide a minimally invasive technique for estimating recent growth rates in marine vertebrates. Hence, as biochemical indices of instantaneous growth are likely temperature and size-dependent, the utility and validation of these indices on marine turtles stocks deserves further study.
